# Thermal Analysis of Plastics Used in the Food Industry

**DOI:** 10.3390/ma15010248

**Published:** 2021-12-29

**Authors:** Małgorzata Majder-Łopatka, Tomasz Węsierski, Artur Ankowski, Kamil Ratajczak, Dominik Duralski, Aleksandra Piechota-Polanczyk, Andrzej Polanczyk

**Affiliations:** 1 Institute of Safety Engineering, The Main School of Fire Service, 52/54 Slowackiego Street, 01-629 Warsaw, Poland; twesierski@sgsp.edu.pl (T.W.); aankowski@sgsp.edu.pl (A.A.); 2 Faculty of Safety Engineering and Civil Protection, The Main School of Fire Service, 52/54 Slowackiego Street, 01-629 Warsaw, Poland; kamil.ratajczak0898@gmail.com (K.R.); apolanczyk@sgsp.edu.pl (A.P.); 3 Institute of Internal Security, The Main School of Fire Service, 52/54 Slowackiego Street, 01-629 Warsaw, Poland; dduralski@sgsp.edu.pl; 4 Jagiellonian University, Faculty of Biochemistry, Biophysics and Biotechnology, Department of Medical Biotechnology, Gronostajowa 7 Street, 30-387 Krakow, Poland; aleksandra.piechota-polanczyk@uj.edu.pl

**Keywords:** differential scanning calorimetry, thermogravimetric, thermal decomposition, polystyrene (PS), polyethylene terephthalate (PET), polypropylene (PP), bond dissociation energy

## Abstract

Fires in landfills, where used plastic packaging waste is discarded, have shown how great a fire hazard these types of materials pose. In this study, the course of thermo-oxidation of samples made of polypropylene (PP), polystyrene (PS), and polyethylene terephthalate (PET) based plastics was determined. Based on an analysis of the dissociation energy of bonds between atoms in a polymer molecule, the mechanisms responsible for the character and course of degradation were observed. It was found that the degradation rate of PP and PS could be a result of the stability of C-H bonds on the tertiary carbon atom. In the case of PS, due to facilitated intramolecular hydrogen transfer, stabilization of hydroperoxide, and formation of a stable tertiary alcohol molecule, the onset of degradation is shifted towards higher temperatures than in the case of PP. Notably, the PP fragmentation occurs to a greater extent due to the easier course of β-scission. In addition, it was found that during a fire, the least amount of heat would be generated by thermo-oxidation of PS-based plastics. This is a result of the formation of a styrene molecule during decomposition that, due to the high stability of bonds in the aromatic ring, escapes from the combustion zone without oxidation. It has been proven that the greatest thermal effect accompanies PET decomposition, during which a phenyl radical is produced, where the C-H bonds break more easily in comparison with the bonds of an intact ring.

## 1. Introduction

Due to plastic’s excellent performance and extensive practicability, global plastic production has reached above 300 million tons [1]. Plastics include diversified groups of materials [2], which makes it impossible to precisely divide plastics according to one criterion [3]. Therefore, different factors are taken into account, i.e., origin, structure, physical properties, and application [4]. Furthermore, the processing and performance properties of plastics, i.e., rheological, mechanical, thermal, structural, morphologic, and optical properties, were highly improved in recent years. This improvement was achieved using various plastic additives such as stabilizers, colorants, plasticizers, fillers and reinforcing fibers, ultraviolet absorbers, antioxidants, and processing aids, including lubricants and flow promoters [5]. Depending on the chemical structure of the main chain, polymers are divided into two groups. The first group consists of polymers with a carbon backbone, e.g., polyethylene, polypropylene, and hydrocarbon polymers with heteroatoms inside groups such as poly (methyl methacrylate). The second group consists of polymers with a heteroatom in the backbone, e.g., oxygen, nitrogen, sulfur, and silicon [6]. Thermosets and thermoplastic materials are highlighted, considering the behavior of the material during heating [7]. Thermoplastic changes into a plastic state when heated and hardens once it has cooled down. However, thermosetting plastics solidify irreversibly when kept at elevated temperatures. Thermoplastic polymers have melting points, whereas thermosetting plastics are relatively rigid solids with a network structure without a melting point. [8]. The thermal decomposition process divides into three basic stages including heating by an external energy source, proper thermal decomposition, and ignition or smoldering of the material, depending on the type of material and the composition of the atmosphere around the sample [9]. Moreover, knowledge about the thermal degradation of polymers, i.e., polystyrene and polyethylene terephthalate, involving the emission of toxic substances, is very important for many commercial applications of these polymers [10,11].

Plastics are used in numerous branches of industry [12]. Packaging accounts for approximately 40% of all plastics produced since the 1950s, of which 41% is used specifically for food or beverages [13]. Food packaging is usually made of plastic due to its lightness, resistance to physical and chemical factors, and low production costs. Most often, it is used in the production of packaging made of plastics based on polyethylene, polypropylene, polyethylene terephthalate, and polystyrene [14,15]. The degradation processes depend on the length of the polymer chain, the additives or the subsequently generated radicals, the temperature, and the environmental composition [16]. However, the reactions of thermo-oxidative degradation are additionally complicated by the participation of oxygen. It has been suggested that the process starts with the formation of hydroperoxide [17]. In most polymers, the rate of this step in the chain reaction determines the overall rate of oxidation [18].

Burning plastic waste in landfill sites increases the intensity of the fire and is the main source of air pollution [19,20]. Most of the time, municipal solid waste containing about 12% plastic is burnt, releasing toxic gases like polycyclic aromatic hydrocarbons, dioxins, furans, and polychlorinated biphenyls into the atmosphere [21,22]. To identify hazardous substances, rescue units around the world use different measuring techniques, including gas chromatography, infrared spectroscopy, ion mobility spectrometry, and electrochemical methods [21,23,24]. Comprehensive knowledge about thermal analysis plays a crucial role in fire risk assessment and determining the processing and recycling conditions of polymers [23]. Thermal analysis has become the primary approach to studying the combustion characteristics of different materials [25,26]. Thermal and thermo-oxidative degradation are the processes that accompany the production, processing, and operation of polymeric materials [27,28]. To propose kinetic models for the thermo-oxidative degradation of plastics, a large body of research has been carried out using various experimental techniques and theoretical approaches [29,30,31,32]. Both calorimetric and optical methods are used [33,34]. Thermogravimetric analysis (TGA) and differential scanning calorimetry (DSC) are two of the conventional methods to investigate the thermal stability of polymers and composites [35,36,37]. Based on these methods, extensive experiments have been conducted on the natural properties of different materials [38,39]. Therefore, the objective of this study was to determine the thermal decomposition mechanism of plastic packaging waste, most commonly stored in landfill sites, that has been set on fire. In this study, a series of TG-DSC experiments for plastics made of polypropylene, polystyrene, and polyethylene terephthalate were conducted under specific temperature conditions to determine the course of thermal decomposition.

## 2. Materials and Methods

In the research, the following five types of plastic packaging materials used in the food industry were applied:

- three sample sets made of polypropylene (PP):
  - PP-1—cookie wrapper;
  - PP-2—cheese tray;
  - PP-3—bread wrapper;

- one sample set made of plastic made of polystyrene (PS):
  - PS-1—cheese cup;

- one sample set made of polyethylene terephthalate (PET):
  - PET-1—a bottle of still water.

Additionally, pure polypropylene (PP-0) was tested. Moreover, according to the manufacturer’s declaration, the PP granulate is used for the production of plastics used in the food industry.

Each time, the weight of the analyzed sample was equal to 10 mg. The samples were analyzed in open alumina crucibles on the TG-DSC sample carrier. The polypropylene-based materials used in the research differed in terms of density (PP-1 0.93 g/cm^3^, PP-2 0.92 g/cm^3^, and PP-3 0.91 g/cm^3^) and flexibility. The most flexible was PP-3, and the least flexible was PP-2. Moreover, the density of PS-1 was equal to 1.05 g/cm^3^, while PET-1 was equal to 1.30 g/cm^3^.

The tested samples were cut out of plastic packages so that they did not contain any imprints. Next, they were placed in the crucible to ensure that the entire bottom surface of the pan was covered.

The TG-DSC STA 499 F5 Jupiter from Netzsch (Selb, Germany) was applied for thermal decomposition analysis. A corundum furnace allowed samples to be heated up to 1100 °C with a heating speed in a range from 0.001 °C/min to 50 °C/min. The constant atmospheric parameters for the tests were maintained by supplying synthetic air. Finally, the received results were analyzed using Proteus^®.^(Selb, Germany).

The dynamic method with constant temperature increases in the range of 35–600 °C was applied for analysis. The temperature increase rate was equal to 10 °C/min. The samples were analyzed in an atmosphere of synthetic air composed of 20% volumetric oxygen at a gas flow equal to 80 mL/min. Moreover, 5.0 purity gases were used in the tests.

The thermal decomposition of the selected plastics was presented as TG and DSC curves. Based on their course, the following parameters were determined using the Proteus^®^ Termal Analysis 8.0.1 software (Netzsch, Selb, Germany):

- the initial temperature of the decomposition process of the sample (beginning of the exothermic reaction);
- the temperature of half mass loss;
- the end temperature of the first step of the decomposition process;
- the mass loss rate for the selected temperatures;
- the energy of endothermic reactions;
- the energy of exothermic reactions.

## 3. Results

Based on the experimental analysis, it was observed that packages made of plastic used for food packaging do not pose a risk in everyday use because their decomposition temperature significantly exceeds the temperature of their use. It has been shown how thermal decomposition proceeds in the presence of oxygen of plastics based on various polymers at a heating rate of 10 °C/min. Furthermore, it was observed that substances added to the polymers might slow down or accelerate the process of plastic thermal decomposition. It has also been shown that the structure of the material has an impact on the amount of energy that needs to be provided to make specific changes in the material.

### 3.1. TG DSC Analysis of Decomposition Process of Plastics Made of Polypropylene

**Figure 1:**
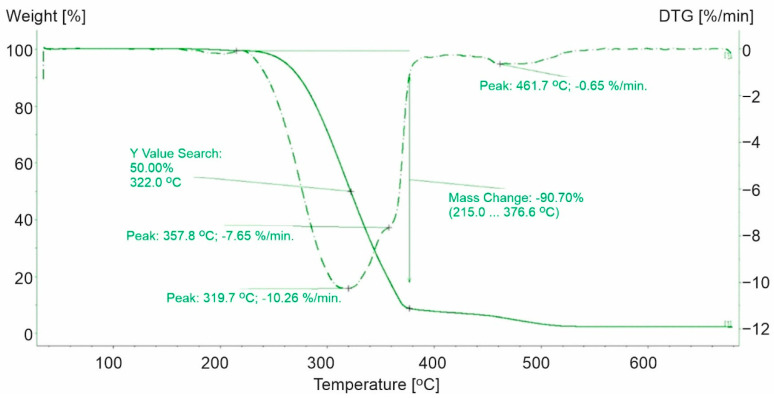
TG and DTG curves for PP-1. The green line presents TG curve, while the green dot line presents DTG curve.

**Figure 2:**
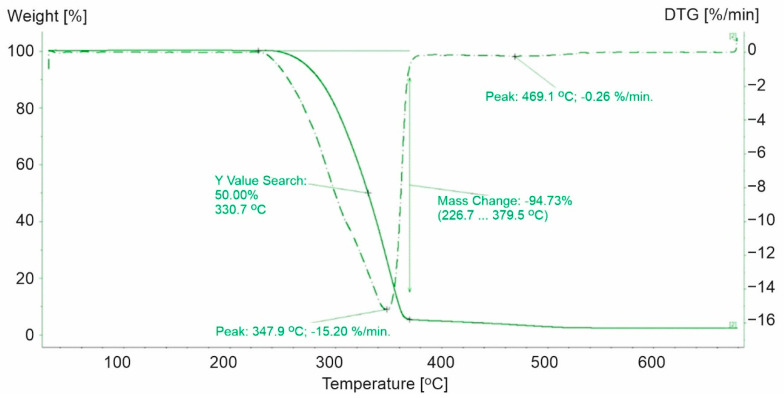
TG and DTG curves for PP-2. The green line presents TG curve, while the green dot line presents DTG curve.

**Figure 3:**
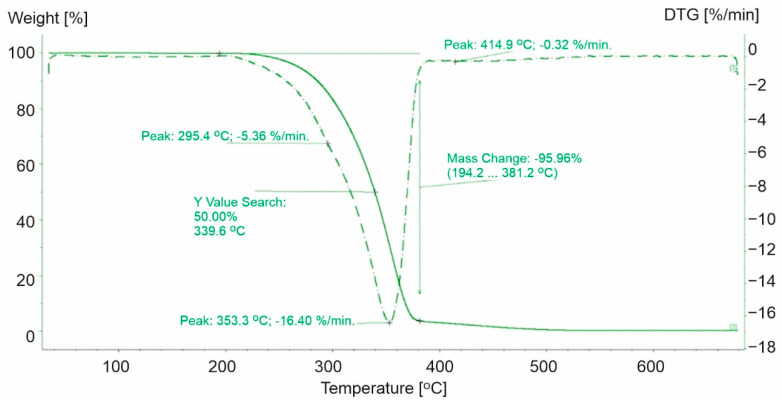
TG and DTG curves for PP-3. The green line presents TG curve, while the green dot line presents DTG curve.

The decomposition of polypropylene plastics was characterized by a two-step process (Figure 1, Figure 2 and Figure 3). For the polypropylene samples, the mass loss for the first step was not a constant value. Weight loss was noted in the first stage from 90.7% (PP-1) to 95.96% (PP-3). PP-0 indicated similar mass loss at the first stage of decomposition to PP-3 (a 96.4% similarity). Furthermore, the highest maximum rate of weight loss for plastics made of polypropylene was recorded for PP-3 (16.4%/min at 353.3 °C) and the lowest for PP-1 (10.26%/min at 319.7 °C). While for PP-0, it was higher and reached 24.01%/min at 291.1 °C.

**Figure 4:**
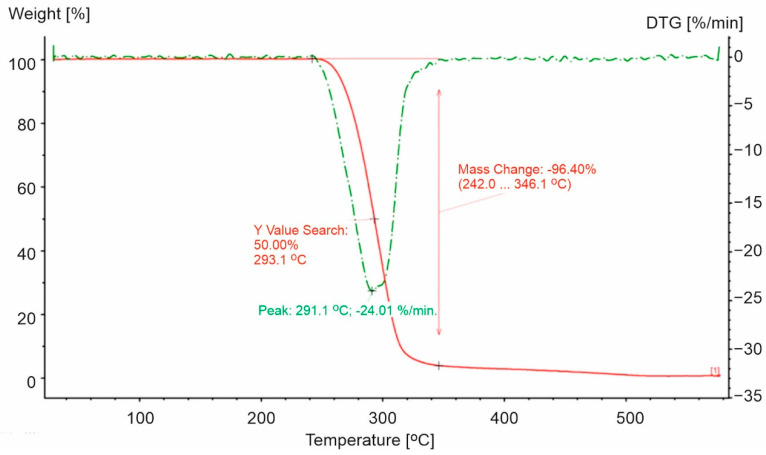
TG and DTG curves for PP-0 in an oxidizing atmosphere (20% O_2_). The red line presents TG curve, while the green dot line presents DTG curve.

The second step of the decomposition process was characterized by a lower dynamic. The highest decomposition rate was observed for PP-1 (0.65%/min at 461.7 °C) (Figure 1), while 0.26%/min (Figure 2) and 0.32%/min (Figure 3) were observed for PP-2 and PP-3, respectively. The identified differences in weight loss and decomposition rates confirmed the influence of additional substances on the plastic’s decomposition process. The course of the TG and DTG curves for the PP-1 sample compared to the PP-0 sample (Figure 4) indicated a significant share of additives in the mass of the analyzed samples. It has been found that polypropylene-based plastics have wider temperature ranges for thermal decomposition than PP-0 homopolymers. The first stage of decomposition takes place in the temperature range of PP (242–346 °C). The results obtained were consistent with those obtained by Saikrasun and Saengsuwa [30].

The lowest decomposition temperature was observed for the PP-3 sample (194.2 °C), and the highest for the PP-2 sample (226.7 °C) (Table 1), while the decomposition temperature for PP-0 was higher at approximately 50 °C and 20 °C, respectively. Such decomposition temperatures were a result of the copolymeric structure of the material. For instance, the PP-3 sample was characterized by greater plasticity compared to the other samples, which was related to the shift of the initial temperature of the thermal decomposition towards lower values. These observations confirmed the results of the research conducted by Chun et al., who found that the structure of the material is an important factor in determining the course of thermal destruction [3].

Moreover, the first step of the thermal decomposition of the tested plastics ended at higher temperatures compared to PP-0 (Table 1). It was noticed that additives increased the limits of thermal decomposition and decreased the dynamics of the process.

Moreover, it was observed that endothermic changes occurring at about 165 °C were internal changes not related to the sample mass change and emission of products into the atmosphere. The recorded peak related to the melting process had a maximum temperature over 4 °C lower than that indicated in the study by Wong and Lam [36]. Similarly, an almost constant melting temperature of about 165 °C for PP was found by Spicker et al. (2019) [40]. Additionally, Brachet et al. (2007) and Navarro et al. (2012) observed the melting point for PP at 160 °C or in a range from 164.2 °C to 166.1 °C, respectively [41,42]. Moreover, Paik and Kar have studied the effect of particle size on the kinetics of thermal degradation for PP. They found that with a change in the particle size of PP, the thermal degradation and diffusion mechanism of degradation products are different even at a constant heating rate [43].

**Figure 5:**
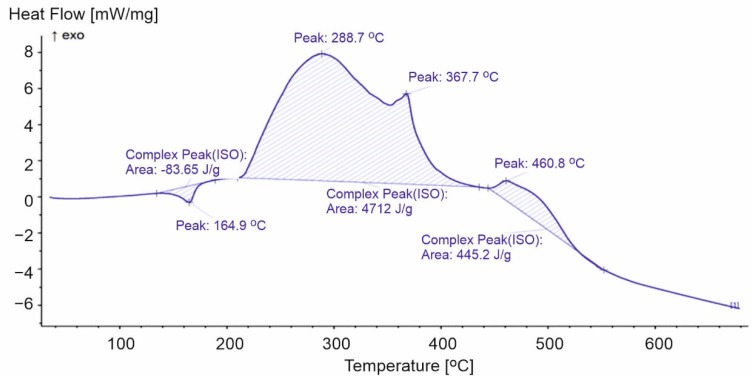
DSC curve for PP-1.

**Figure 6:**
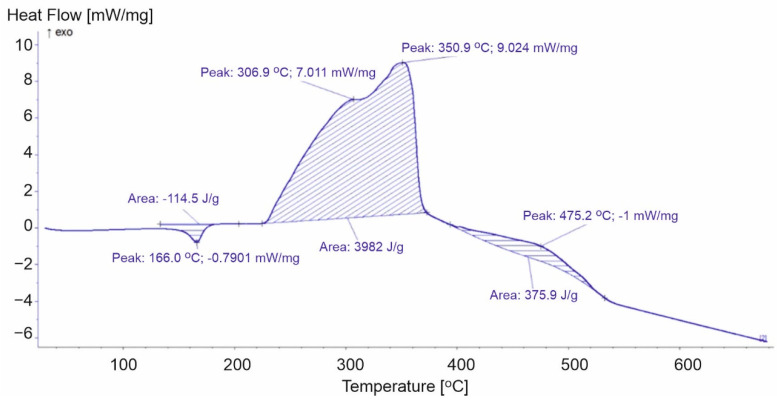
DSC curve for PP-2.

**Figure 7:**
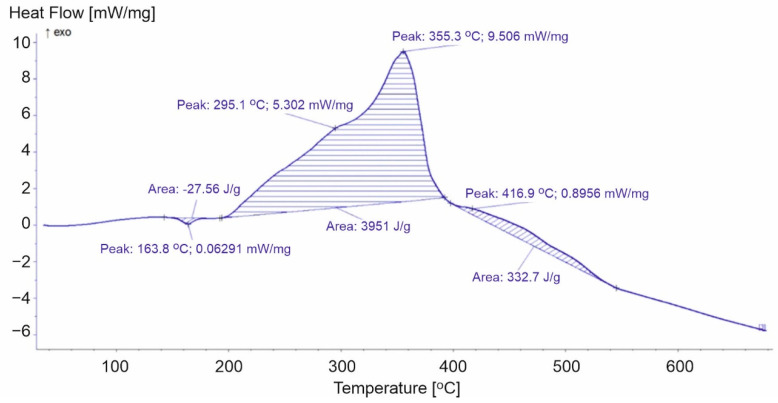
DSC curve for PP-3.

Polypropylene film is more ductile than the other products and, therefore, requires less energy for the phase transition. The energy of the PP-3 sample transformation (27.56 J/g) was much lower compared to the other analyzed samples (PP-1 83.65 J/g and PP-2 114.5 J/g) (Figure 5 and Figure 6). After the endothermic peak related to the melting of the sample at a temperature above 200 °C, exothermic peaks associated with the oxidation of the first- and second-order radicals and propene formed during thermal decomposition were recorded [18]. The first exothermic transformation of the PP-2 and PP-3 samples released similar energy (3982 J/g and 3951 J/g, respectively), while for PP-1, it was equal to 4712 J/g. The second exothermic reactions correspond to another loss of mass. In the case of the PP-1 sample, during the second stage of decomposition, we recorded the highest loss of weight from all analyzed PPs. The transformation released energy at a level equal to 445.2 J/g. Lower values equal to 375.9 J/g and 332.7 J/g, respectively (Figure 6 and Figure 7) were noticed for PP-2 and PP-3. The additives used during the production of the PP-1 material increased the energy released during the entire decomposition process compared to the PP-2 and PP-3 samples.

During the first stage of decomposition, two peaks were recorded. The higher of these peaks in PP-2 and PP-3 corresponded to the temperature of the maximum rate of mass loss. For PP-2, peak number three appeared at 350.9 °C, and the fastest mass loss occurred at 347.9 °C. While for PP-3, these values were equal to 355.3 °C and 353.3 °C. In the case of the PP-1 sample, no such relationship was observed. The maximum speed of mass loss was recorded at 319.7 °C, an exothermic peak at 288.7 °C. Peak number four corresponded to the maximum rates of mass loss during the second stage of decomposition. The analysis of the results of the PP-2 and PP-3 samples allowed the correlation of the reaction energy of exothermic polypropylene materials with the rate of weight loss of the samples. However, the course of the DSC curve for the PP-1 samples did not allow for unambiguous confirmation of this relationship (Table 2).

### 3.2. TG DSC Analysis of Decomposition Process of Plastics Made of Polystyrene

**Figure 8:**
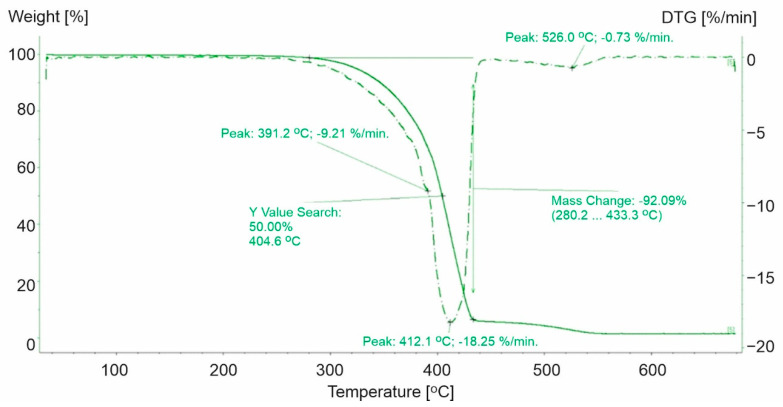
TG and DTG curves for PS-1. The green line presents TG curve, while the green dot line presents DTG curve.

The analysis of the TG and DTG curves for PS-1 (Figure 8) indicated that thermal decomposition took place in two steps. The temperature range for the first step was equal to 280.2–433.3 °C (the remaining mass after the test was equal to 1.6% of the original sample mass) (Figure 8). In contrast, PS-0 was characterized by a one-step decomposition process and took place from 260 °C to 424 °C with a maximum speed of 392 °C [37].

For the second step of the decomposition process for PS-1, the mass loss rate reached 0.73 °C/min at a temperature equal to 526 °C (above the temperature of the end of the decomposition process of pure polystyrene) (Figure 8). The differences during the thermal decomposition of PS-1 and PS-0 were caused by the presence of additives used in the processing of the materials. Their presence was characterized by the irregular course of the sample mass loss rate and the two-step process. It was observed that the application of other substances increased the initial temperature of the decomposition process and delayed the time necessary to reach the maximum decomposition rate (Table 3).

**Figure 9:**
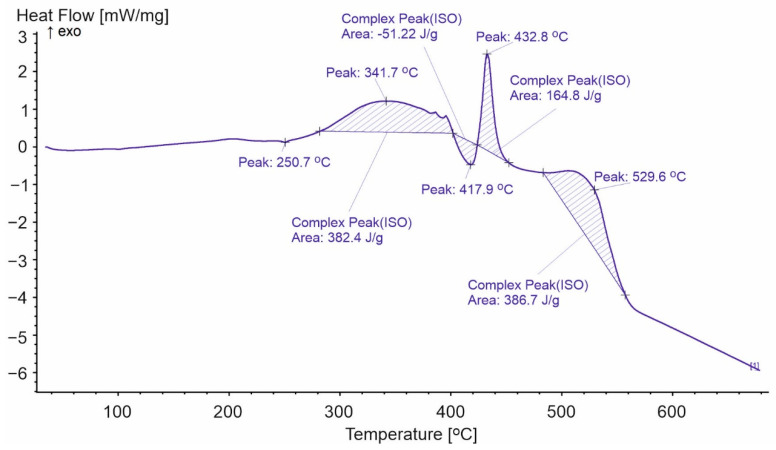
DSC curve for PS-1.

Moreover, the DSC curve indicated that during the loss of weight of the analyzed sample, exothermic and endothermic changes appeared. Sample decomposition was preceded by a low energy endothermic reaction at a maximum peak equal to 250 °C. The conducted analysis indicated that over 92% of sample weight loss decomposed at the first stage, where the maximum speed of decomposition was observed at 412 °C. Our observations align with Wong et al., who showed that at 420 °C polystyrene decomposed completely within 10 min [44]. The mechanism of thermal decomposition of polystyrene is complex and includes reaction initiation at the ends of polymers chains, depolymerization, oxidation, an intramolecular shift of the hydrogen atom, and kinetic chain termination by recombination or disproportionation (dismutation) [31]. These processes run both with absorption and the transfer of energy to the surroundings (Figure 9).

### 3.3. TG DSC Analysis of Decomposition Process of Plastics Made of Polyethylene Terephthalate

**Figure 10:**
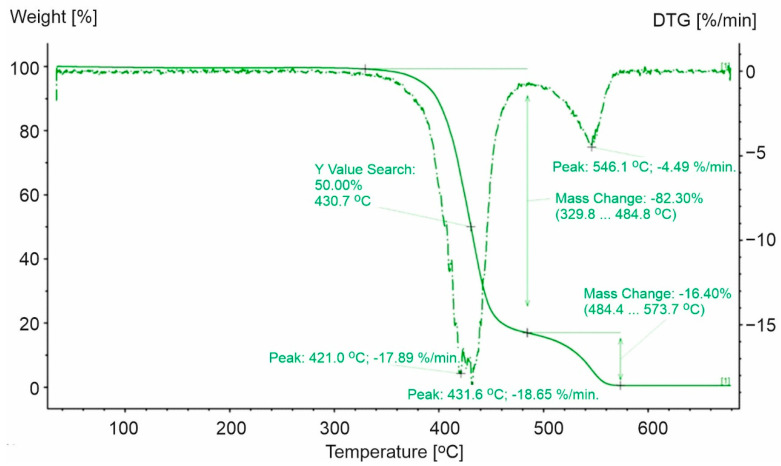
TG and DTG curves for PET-1. The green line presents TG curve, while the green dot line presents DTG curve.

The initial temperature of the decomposition process of the tested sample was much lower (329.8 °C) compared to PET-0 (390 °C) (Figure 10). In the study of PET-1, the beginning of weight loss was correlated with the initial temperature of internal changes based on the DSC results. Moreover, in the study of PET-0, this value was determined based on the loss of a specific weight. A temperature of 573.7 °C indicated the end of the decomposition process of the analyzed sample, which coincides with the result of PET-0 [15,28].

The process of thermal decomposition of PET-1 and PET-0 took place in two steps. The weight loss in the first step was equal to 82.33% for PET-1 and in the second step to 16.4% of its weight (Figure 10), while the loss for PET-0 was equal to 78.5% and 21.1%, respectively (Table 4). The differences proved that the influence of the additives present in the material affected the course of the decomposition process.

**Figure 11:**
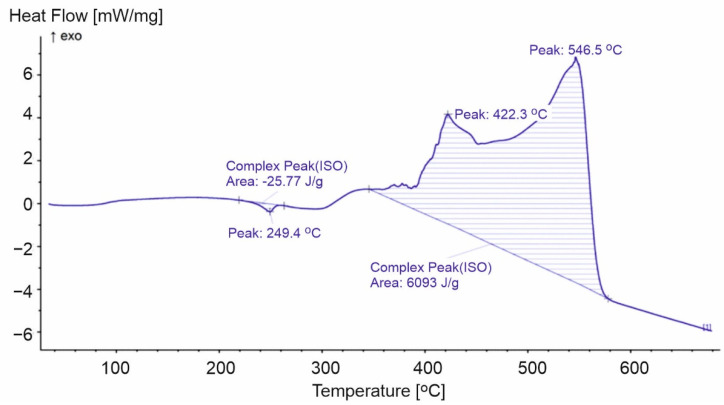
DSC curve for PET-1.

The comparison of the TG and DTG curves in Figure 10 with the calorimetric curves shown in Figure 11 indicated that the endothermic transformation observed at 249.4 °C was not related to weight change. However, an internal transformation took place with the absorption of energy from the environment. Endothermic transformation initiates thermal decomposition combined with weight change, which turns into an exothermic transformation. The exothermic peaks at 422.3 °C and 546.5 °C correlated with the temperature of the maximum rate of weight loss (Figure 10).

## 4. Discussion

**Figure 12:**
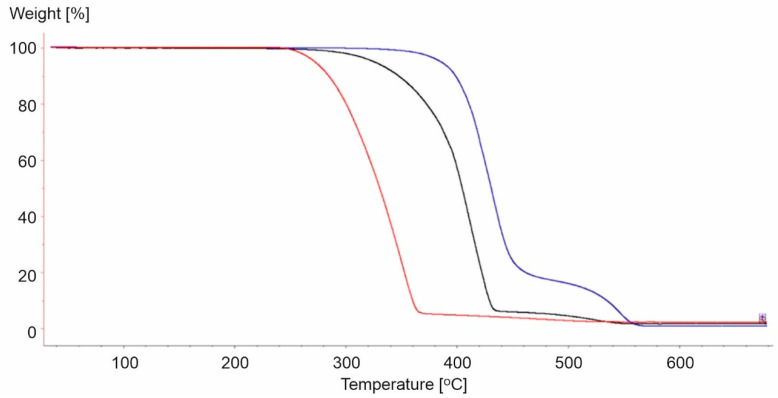
TG curves for PP-3 (red line), PS-1 (black line), and PET-1 (blue line).

It was observed that the thermal decomposition of the analyzed materials began at different temperatures (Figure 12). The lowest temperature (194 °C) was observed for the PP-3 sample, while the highest was for PET-1 (329.8 °C). Furthermore, thermal destruction of PS-1 began at 280 °C.

Therefore, the loss of half the weight for PP-3 was observed at a lower temperature (340 °C) compared to 405 °C and 431 °C for PS-1 and PET-1, respectively. Furthermore, PET-1 presented the most distinct course of the second stage of decomposition, which ended at 574 °C.

In order to refer to the obtained experimental results, a review was carried out of the values of bond dissociation energies available in the literature to propose a dissociation mechanism of particular polymer molecules with respect to structural similarities of the selected organic compounds described in [45].

**Figure 13:**
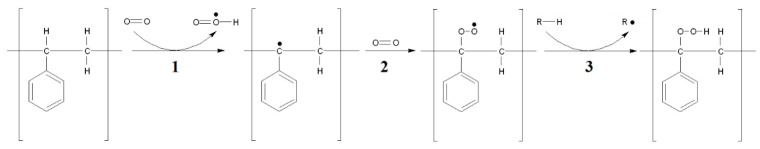
The possible scheme of formulation of tertiary peroxide by oxygen reaction using PS chain. The following symbols were applied: 1—an attack of an oxygen molecule on a PS molecule with formation of a radical; 2—an attack of an oxygen molecule on a radical located on a tertiary carbon atom; 3—transfer of hydrogen from cleavage of the C-H bond of the polymer structure with formation of a hydroperoxide.

The initial step in the formation of oxygen bonds is a reaction in which a radical on the carbon chain is created [46]. For both PP and PS, the formation of a radical, by reaction with oxygen or pyrolysis, will be more favored on the 3° carbon atom than on the 2° carbon atom [47]. The reaction with O_2_ will lead to the formation of a tertiary hydroperoxide (Figure 13). The formation of the radical on PS will be easier than on PP due to the lower dissociation energy (BDE-bond dissociation energy) of the C-H bond in position *β* in relation to the aromatic ring. This can be illustrated by the example of compounds with a similar environment, 2-Phenylpropane and 2-Methylpropane for PS and PP, respectively. BDEs for 2-Phenylpropane is DH_0_(C-H) = 348 kJ/mol, while for 2-Methylpropane DH_0_(C-H) = 400.4 kJ/mol [45]. Furthermore, the hydrogen bond breaking to form a radical on the 2° carbon atom costs ca. 10 kJ/mol more (e.g., for n-Propane, the C-H bond breaking on the central carbon atom DH_0_(C-H) = 410.5 kJ/mol) [45].

**Figure 14:**
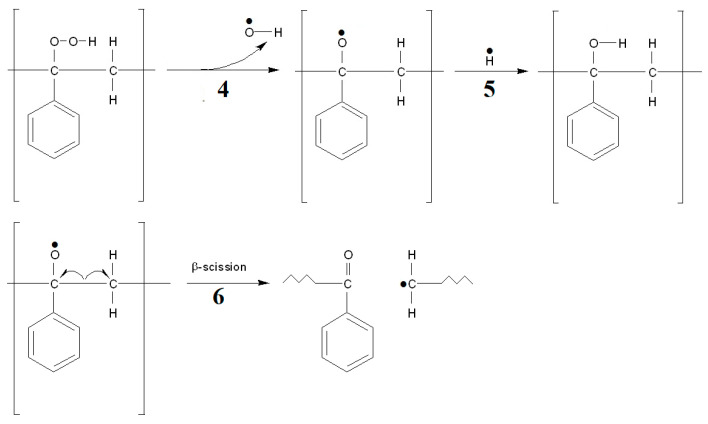
The proposed degradation pathways of tertiary hydroperoxide. The following symbols were applied: 4—decomposition of the hydroperoxide on the O-O bond with departure of the OH radical; 5—reaction of the resulting tertiary oxide radical with formation of alcohol; 6—decomposition at the position of the tertiary oxide radical.

The presence of hindered phenols (PhOH) with strong reducing properties can significantly slow down the polymer degradation process in the initial stage [47,48,49]. It is presumed that this could be due to the transfer of the hydrogen atom to the phenoxyl group, which causes stabilization of the peroxyl radical by the formation of hydroperoxide. In Figure 13, intramolecular hydrogen transfer in the PS structure is presented in reaction 3. In contrast to PP, polystyrene could form some phenolic compounds during the degradation process. However, it is rather unlikely, especially at the initial stage of the reaction. Breaking the C-H bond in the aromatic ring is an energy expenditure of around 472.2 kJ/mol, and hence, it is much more than breaking the bond in the aliphatic chain [45]. Therefore, in the analyses of PS decomposition products, no phenolic compounds are observed, and practically, the dominant oxygen compound containing an aromatic ring is benzaldehyde [11,50,51]. Stabilizing hydrogen transfer directly from the structure of PS (Figure 13, reaction 3) will be, on the other hand, possible by breaking the C-H bond from other sides of the polymer molecule, with the easiest breaking point in position *β* relative to the aromatic ring. Due to the previously mentioned bond dissociation energy, this is likely to be easier in PS than PP. The facilitated transfer and formation of hydroperoxide may be the reason for the much higher onset temperature of PS decomposition. The formation of hydroperoxide inhibits the decomposition of the carbon chain in position *β* (*β* scission) if hydrogen transfer is possible to form the alcohol, as shown in Figure 14. Moreover, this transfer, as mentioned earlier, is easier in the PS molecule.

The thermal effect observed in this study for PS is less intense than that for PP. The decomposition of delocalized bonds between carbon atoms of the aromatic ring requires a very high energy input (DH_0_(C-C) = 518 kJ/mol), which may explain the presence of an endothermic peak with its maximum at 417 °C. Hence, it is much easier for the C-C bond to break down in position *β* in relation to the ring. This causes the separation of a styrene molecule, which leaves the combustion zone non-oxidized, contrary to the small and easily volatile vapors of the resulting propene. Therefore, the DSC curves indicate that the amount of heat recorded for exothermic processes of PP is more than 4000 J/g and for PS less than 1000 J/g. This observation is confirmed by the studies conducted by Zhu et al., who has identified styrene as the predominant product under both oxidative and pyrolysis conditions in the presence of nitrogen [51]. Peterson et al. indicated that 40% of the products are styrene during thermal PS decomposition [18]. The decomposition of the C-C bond with the formation of styrene requires relatively little energy. For 2-Phenylpropane with a local bond system analogous to PS, the dissociation energy of the C-C bond in position *β* relative to the ring is only DH_0_(C-C) = 318 kJ/mol [45]. Hence, dissociation at this position is relatively easy, which is also confirmed by computer simulations performed for the tristrene molecule [52].

**Figure 15:**
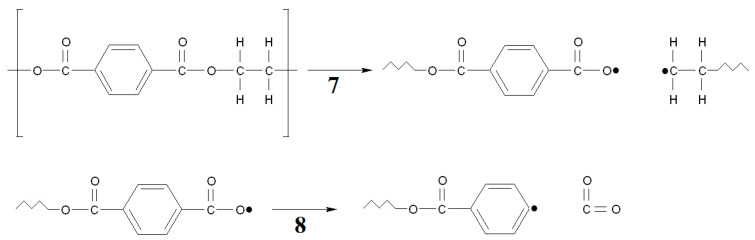
Exemplary degeneration of PET. The following symbols were applied: 7—Homolytic breaking of the C-O bond in PET between the carboxyl and ethylene groups; 8—CO_2_; detachment with formation of phenyl radical.

When comparing PP, PS, and PET, it is worth noting that the process of mass loss starts last in the case of PET. To explain the reason for later degradation, it is worth paying attention to the characteristics of the energy of bonds in PET by comparing it to the energy of breaking bonds in methyl benzoate: breaking the -C-H bond in the PET ethylene chain is more difficult. The energy of the C-H bond in the -CH_3_ group of methyl benzoate is 418.4 kJ/mol, which is higher than in 2-Phenylpropane (DH_0_(C-H) = 348 kJ/mol) and 2-Methylpropane (DH_0_(C-H) = 400.4 kJ/mol). It will, therefore, be more difficult to produce a radical by breaking the C-H bond. Further, even more energy is required to break the C-C bond between the carboxyl group and the aromatic ring, where 436.8 kJ/mol is required. The C-O bond between a methyl group and a carboxyl group breaks more easily and needs 372.8 kJ/mol. It is more difficult to break a single C-O bond in a carboxyl group that requires 413.4 kJ/mol. To determine the weakest point of the molecule, it is worth using the value of energy between carbon atoms of the ethylene group. For ethylene glycol, the value of DH_0_(C-C) is 358.2 kJ/mol, which is clearly less than in the other analyzed structural elements. However, these values are much higher than those required, such as breaking the C-C bond in polystyrene (DH_0_(C-C) = 318 kJ/mol). Hence, the PET structure will break at a higher temperature than PS and PP. When the C-O bond between the carboxyl and ethylene groups is broken, a radical is formed on the oxygen atom of the carboxyl group, which decomposes very easily with the release of CO_2_ and the formation of a radical on the aromatic ring (Figure 15). The C-H bond energy in the phenyl radical (327.6 kJ/mol) is much lower than in benzene (472.2 kJ/mol) [45]. The degradation of the phenyl radical at a high temperature will require much less energy for its transformation than the breakdown of aromatic rings in PS. Therefore, the amount of energy obtained will also be much higher, which is confirmed by PET pyrolysis studies [53]. This is also confirmed by the DSC curves we obtained for PET, where the value of 6093 J/g was obtained, which is even higher than for PP. Thus, there is a high probability that the PET degradation process occurs with more intensive fragmentation than in PS and PP. The course of the process, according to Figure 15, is confirmed by Brems et al. [53].

**Figure 16:**
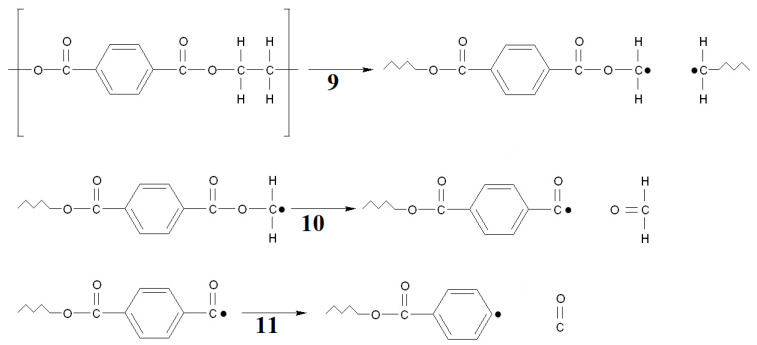
Schematic degeneration of PET by bond breaking at the ethylene element. The following symbols were applied: 9—cleavage of the C-C bond in the ethylene group; 10—formation of a benzoyl radical with a formaldehyde moiety; 11—cleavage of the benzoyl radical with formation of a phenyl radical.

Regarding breaking the C-C bond in the ethylene element, the radical formed on the carbon atom is unstable and can be transformed with the formation of a formaldehyde molecule and a benzoyl radical (Figure 16, reaction 9, 10). For example, only 47 kJ/mol [45] is needed to break the C-O bond in the carboxyl group of the acetic acid radical (*CH_2_COOH). The decomposition of the benzoyl radical also requires a small amount of energy, only 103.3 kJ/mol [45]. This pathway would also result in the formation of a phenyl radical (Figure 16, reaction 11).

Interestingly, a similar sequence of oxidative transformations of PET is observed during photo-oxidation processes following Norrish Type I reaction mechanisms [54].

**Figure 17:**
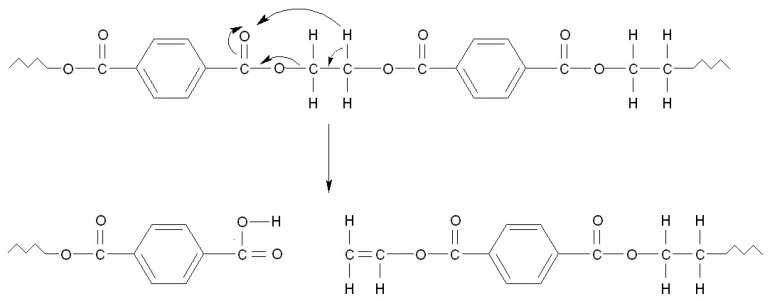
Schematic PET decomposition according to Norrish type reaction II. As a result, during subsequent reactions, terephthalic acid and derivatives containing vinyl group are formed.

Brems et al. also observed the formation of terephthalic acid and vinyl compounds during PET pyrolysis [53], which corresponds to the Norrish Type II reaction with internal hydrogen transfer (Figure 17). In the case of PET, terephthalic acid and benzoic acid may constitute a significant part of post-pyrolytic residues.

The PP samples analyzed in our study were characterized by visible differences in thermal properties, which indicated differences in the structure. The most commonly used industrial propylene polymers, apart from the homopolymer, which is composed only of propylene units, are propylene and ethylene copolymers characterized by higher mechanical resistance [55,56,57]. The number of tertiary carbon atoms on the homopolymer will be much higher than the copolymer containing ethylene units. Only secondary carbon atoms will be present on the ethylene elements. Therefore, considering that the decay on the tertiary atom is easier than on the secondary atom, which is a result of the lower bond energy, we observed faster degradation of PP homopolymers (propylene homopolymer). This can be observed following the decomposition rate of the samples used in our study. The 50% weight loss temperature was the highest for PP-0 that was a pure homopolymer (T = 293 °C), while for PP-1, PP-2, and PP-3, the temperatures were equal to 322 °C, 330.7 °C, and 339.6 °C, respectively. A similar dependence was observed for the temperature of the maximum rate of weight loss. Moreover, the maximum rate of weight loss was also observed for the PP-0 sample (Table 1). Hence, we speculate that the PP-1, PP-2, and PP-3 samples were copolymers in which, apart from the PP, the PE part was present. A similar observation was made for PS-0 and PS. However, the situation was different in the case of PET-0 and PET-1. A comparison of the decomposition onset temperature and the degree of weight loss in the first decomposition step indicated that PET-1 decomposed more easily. As indicated above, the weakest point of the PET molecule was the C-C bond between the carbon atoms of the ethylene group. The incorporation of units composed of more methylene units (-CH_2_-) in a part of the copolymer molecule, instead of the ethylene group, increases the share of the weakest aliphatic C-C bonds in the polymer molecule and facilitates its decomposition [53].

## 5. Conclusions

Plastic packaging still makes up a significant proportion of the waste collected in municipal landfills and poses a major risk in the event of fires. The thermal stability of these materials depends primarily on the type of polymer, the additives used, and the structure. Of the plastics subjected to testing, the PP plastics were found to have the lowest thermal stability, while the PET thermoplastics had the highest. The rate of degradation of PP and PS may be due to the relative weakness of C-H bonds on the tertiary carbon atom. The decomposition of this bond enables the stabilization of hydroperoxide, which with a sufficiently easy detachment of the hydrogen atom and intramolecular transfer, transforms into stable tertiary alcohol. Due to the ease of detachment of the hydrogen atom from the benzylic carbon atom, this process has a preferred progression for PS. Hence, PS degradation will occur at higher temperatures than PP. The low intramolecular transfer of hydrogen causes an increased amount of β-scission, causing fragmentation of the molecule.

Additionally, it was found that the smallest amount of heat during a fire would be decidedly generated by thermo-oxidation of PS-based plastics. This is due to the formation of a styrene molecule during heating, which is due to the high stability of bonds in the aromatic ring and volatility escaping from the combustion zone without oxidation. The PET, despite having an aromatic ring in its structure, also releases a very large amount of heat during the thermo-oxidation process. The amount is even higher than in the case of PP. Analyzing the potential mechanism suggests that during decomposition, a phenyl radical is produced, and its decomposition is much easier than that of the neutral aromatic ring. Therefore, fragmentation of PET molecules occurs more intensively than in the case of PS.

The PP-based plastics were found to have a lower maximum rate of weight loss and a shift in the 50% weight loss temperature towards lower temperature values compared to the base polymer and polystyrene and PET-based plastics. 

Additionally, results show that the PP-1, PP-2, and PP-3 samples can consist of PP and PE copolymers, which was supported by the decomposition rates of the samples analyzed in our study. The 50% weight loss temperature and the temperature of the maximum rate of weight loss were lowest for PP-0, while the rate of weight loss was highest for PP-0. This indicated a faster decomposition of pure PP due to a greater proportion of tertiary carbon atoms compared to the copolymer-containing ethylene units. A similar observation was made for PS-0 and PS. 

The PS-based packaging was found to start decomposing at a higher temperature than pure polystyrene. Unfortunately, the opposite trend was recorded for PET packaging. In the case of PET, faster degradation was observed for PET-1, which may indicate the presence of longer aliphatic elements in the molecule containing more methylene units, which increased the probability of breaking the C-C bond compared to PET containing an ethylene fragment.

## Figures and Tables

**Table 1:** The most important parameters for the thermal decomposition process for PP-0, PP-1, PP-2, and PP-3 samples.

Parameters of Thermal Decomposition	PP-0	PP-1	PP-2	PP-3
Initial decomposition temperature [°C]	242.0	215.0	226.7	194.2
End temperature of 1st step of decomposition [°C]	346.1	376.6	369.5	381.2
Loss of weight for 1st step of decomposition [%]	96.4	90.7	94.7	95.9
Temperature of loss of 50% of weigh [°C]	293.1	322.0	330.7	339.6
Maximum rate of weight loss [%/min]	24.01	10.26	15.2	16.4
Temperature of maximum rate of weight loss [°C]	293.1	319.7	347.9	353.3
Maximum rate of weight loss for 2nd stage [%/min]		0.65	0.26	0.32
Temperature of maximum rate of weight loss for 2nd stage [°C]		461.7	469.1	414.9

**Table 2:** Selected parameters for the DSC analysis for polypropylene.

Parameters of Calorimetric Analysis	PP-1	PP-2	PP-3
1st peak [°C]	164.9	166.0	163.8
2nd peak [°C]	288.7	306.9	295.1
3rd peak [°C]	367.7	350.9	355.3
Energy of the 1st transformation [J/g]	83.65	114.5	27.56
Energy of the 2nd transformation [J/g]	4712	3982	3951
Energy of the 3rd transformation [J/g]	445.2	375.9	332.7

**Table 3:** Selected parameters for the thermal decomposition process for polystyrene.

Parameters of Thermal Decomposition	PS-0	PS-1
Initial decomposition temperature [°C]	260.0	280.2
End temperature of 1st stage of decomposition [°C]	424.0	433.3
Loss of weight for 1st stage of decomposition [%]	98.0	92.1
Temperature of loss of 50% of weight [°C]	-	404.6
Maximum rate of weight loss [%/min]	-	18.2
Temperature of maximum rate of weight loss [°C]	392.0	412.1
Maximum rate of weight loss for 2nd stage [%/min]	-	0.7
Temperature of maximum rate of weight loss for 2nd stage [°C]	-	526.0
Residual mass [%]	2.0	1.4

**Table 4:** Selected parameters for the thermal decomposition process for PET-0 and PET-1.

Parameters of Thermal Decomposition	PET-0	PET-1
Initial decomposition temperature [°C]	390.0	329.8
End temperature of 1st stage of decomposition [°C]	-	484.8
Loss of weight for 1st stage of decomposition [%]	78.5	82.3
Temperature of loss of 50% of weight [°C]		430.7
End temperature of 2nd stage of decomposition [°C]	575.0	573.7
Loss of weight for 2nd stage of decomposition [%]	21.1	16.4
Maximum rate of weight loss [%/min]	-	18.65
Temperature of maximum rate of weight loss [°C]	-	431.6
Maximum rate of weight loss for 2nd stage [%/min]	-	4.49
Temperature of maximum rate of weight loss for 2nd stage [°C]	-	546.1
Residual mass [%]	0.4	1.3

## Data Availability

Data is available on request from the corresponding author.
